# Complete mitochondrial genome and phylogenetic analysis of *Mastax latefasciata* Liebke 1931 (Insecta: Coleoptera: Carabidae)

**DOI:** 10.1080/23802359.2022.2151829

**Published:** 2022-12-12

**Authors:** Yu Bai, Lin Ye, Kang Yang, Xuyuan Gao

**Affiliations:** a College of Mathematics & Information Science, Guiyang University, Guiyang, China; b College of Biology and Environmental Engineering, Guiyang University, Guiyang City, China; c Guangxi Key Laboratory of Biology for Crop Diseases and Insect Pests, Institute of Plant Protection, Guangxi Academy of Agricultural Sciences, Nanning, China; d Key Laboratory of Green Prevention and Control on Fruits and Vegetables in South China Ministry of Agriculture and Rural Affairs, Institute of Plant Protection, Guangxi Academy of Agricultural Sciences, Nanning, China

**Keywords:** *Mastax latefasciata*, Carabidae, mitochondrial genome, phylogenetic analysis

## Abstract

The genus *Mastax* Fischer von Waldheim 1827 belongs to the family Carabidae. Specimens of adult *Mastax latefasciata* Liebke, 1931 were collected from Yājì Hill, Huáihuà City, Húnán Province, China. The complete mitochondrial genome (GenBank accession number ON674050.1) of *M. latefasciata* was sequenced, annotated, and characterized. The results showed that it was a circular DNA molecule of 16,735 bp with 81.07% AT content and comprised 13 protein-coding genes (PCG), 22 tRNA genes, 2 rRNA genes, and 1 control region. The PCGs were initiated using typical ATN (Met) and TTG (Met) start codons and terminated using typical TAN stop codons. The phylogenetic position of *Mastax* within the Carabidae was first evaluated using complete mitogenomes, and the results showed that it was close to *Cicindela anchoralis* and *Manticora tibialis*.

The genus *Mastax* Fischer von Waldheim 1827 belongs to the tribe Brachinini of the family Carabidae (Liang and Yu 2004), and eight species have been recognized in China (Liang and Yu 2004). Jedlička in Eastern Asia provided a key for *Mastax latefasciata* Liebke, 1931 (Liebke 1931; Liang and Yu 2004), which is the basal half of the elytra with one yellow square band and a band width of approximately two-fifths the length of the elytra (Liang and Yu 2004; Figure 1). At present, the nucleotide database of the National Center for Biotechnology Information (NCBI) has not publicly published the mitochondrial information of the genus *Mastax*, with the exception of the 16S ribosomal RNA (rRNA) gene of *M. formosana. M. latefasciata* is relatively common and readily available in China. In this study, the complete mitochondrial genome (mitogenome) of *M. latefasciata* was first sequenced, annotated and characterized, which would have significance for contributing to the research on phylogenetic position of the genus *Mastax*.

**Figure 1.**
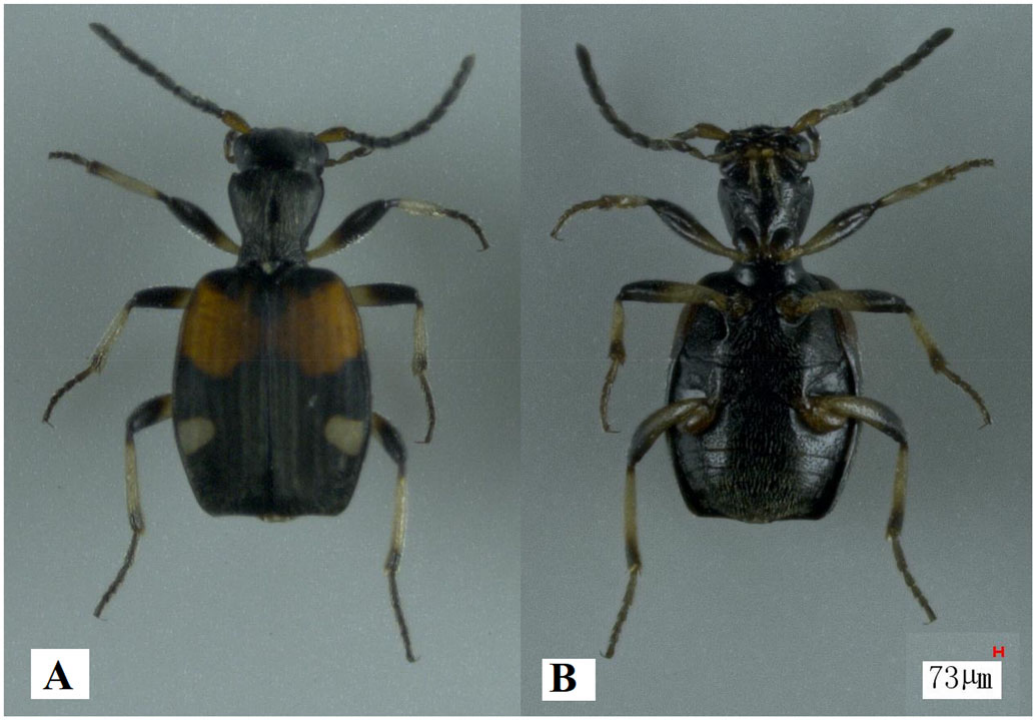
Species reference image of *Mastax latefaciata*. (A) the ventral view of *M. latefaciata*; (B) the dorsal view of *M. latefaciata. M. latefaciata* was imaged using VHX-2000 digital microscope system.

Specimens of adult *M. latefasciata* were collected from Yājì Hill (110.55° N, 27.85° E), Zhòngxià Village, Xùpǔ County, Huáihuà City, Húnán Province, China, on April 24, 2022, and deposited in the Insect Collection of Institute of Plant Protection, Guangxi Academy of Agricultural Sciences (http://www.gxaas.net/s.php/zwbhyjs/, Xuyuan Gao, gxy@gxaas.net) under the voucher number GIPP-20220424-001.

Genomic DNA was isolated using the Qiagen DNeasy Blood and Tissue Extraction kit (Qiagen, Germantown, MD, USA) and subjected to paired-end sequencing (2 × 150 bp) of 300 bp inserts using an Illumina NovaSeq 6000 platform (Illumina, Inc., San Diego, CA, USA). The obtained raw reads were filtered to obtain clean reads using fastp v0.23.2 (https://github.com/OpenGene/fastp) (Chen et al. 2018). The quality control (QC) standards of reads from DNA were: (1) Trimming adapter sequences with >6 bases, (2) Removing reads with >0 unidentified nucleotides (N), (3) Removing reads with >20% bases with Phred quality < Q30, (4) Removing reads with <150 bases. We obtained approximately 30.13 Gb of clean high-quality data from the raw data of 35.80 Gb. The complete circular mitogenome (GenBank accession number: ON674050.1) of *M. latefasciata* was assembled *de novo* using NOVOPlasty v4.3.1 (https://github.com/ndierckx/NOVOPlasty) (Dierckxsens et al. 2017) with default parameters and the mitogenome of *Harpalus sinicus* (GenBank accession number: MN310888.1/NC_045094.1) (Yu et al. 2019) used as a seed sequence, which was found to be 16,735 bp (nucleotide composition: 40.34% A, 40.73% T, 7.52% C, and 11.41% G) in length with 81.07% AT content. The AT-skew [(A − T)/(A + T)] and GC-skew [(G − C)/(G + C)] of the sequence were estimated to investigate the nucleotide composition bias using Perna and Kocher’s formula (Perna and Kocher 1995). The AT and GC skews of the major strand of the mitogenome were estimated to be −0.00479 and 0.20581, respectively. The mitogenome of *M. latefasciata* was initially annotated using GeSeq v2.03 (https://chlorobox.mpimp-golm.mpg.de/geseq.html) (Tillich et al. 2017), using the third-party software tRNAscan-SE v2.0.7 (Chan and Lowe 2019), ARWEN v1.2.3 (Laslett and Canbäck 2008), BLAT v36 × 7 (Kent 2002) with the mitogenome of *Harpalus sinicus* (MN310888.1/NC_045094.1) as a reference. In addition, the start and stop codons of protein-coding genes (PCGs) were corrected manually using the genomes of *Harpalus sinicus* (MN310888.1/NC_045094.1) (Yu et al. 2019), *Promethis valgipes valgipes* (Bai et al. 2021), *Tenebrio obscurus* (Bai et al. 2018), and *Zophobas atratus* (Bai et al. 2019) as references. The mitogenome of *M. latefasciata* comprises 13 PCGs, 1 control region (CR), 22 tRNA genes, and 2 rRNA genes. The order and orientation of the genes were determined and drawn (Figure 2) using the OGDRAW web server (https://chlorobox.mpimp-golm.mpg.de/OGDraw.html) (Greiner et al. 2019).

**Figure 2.**
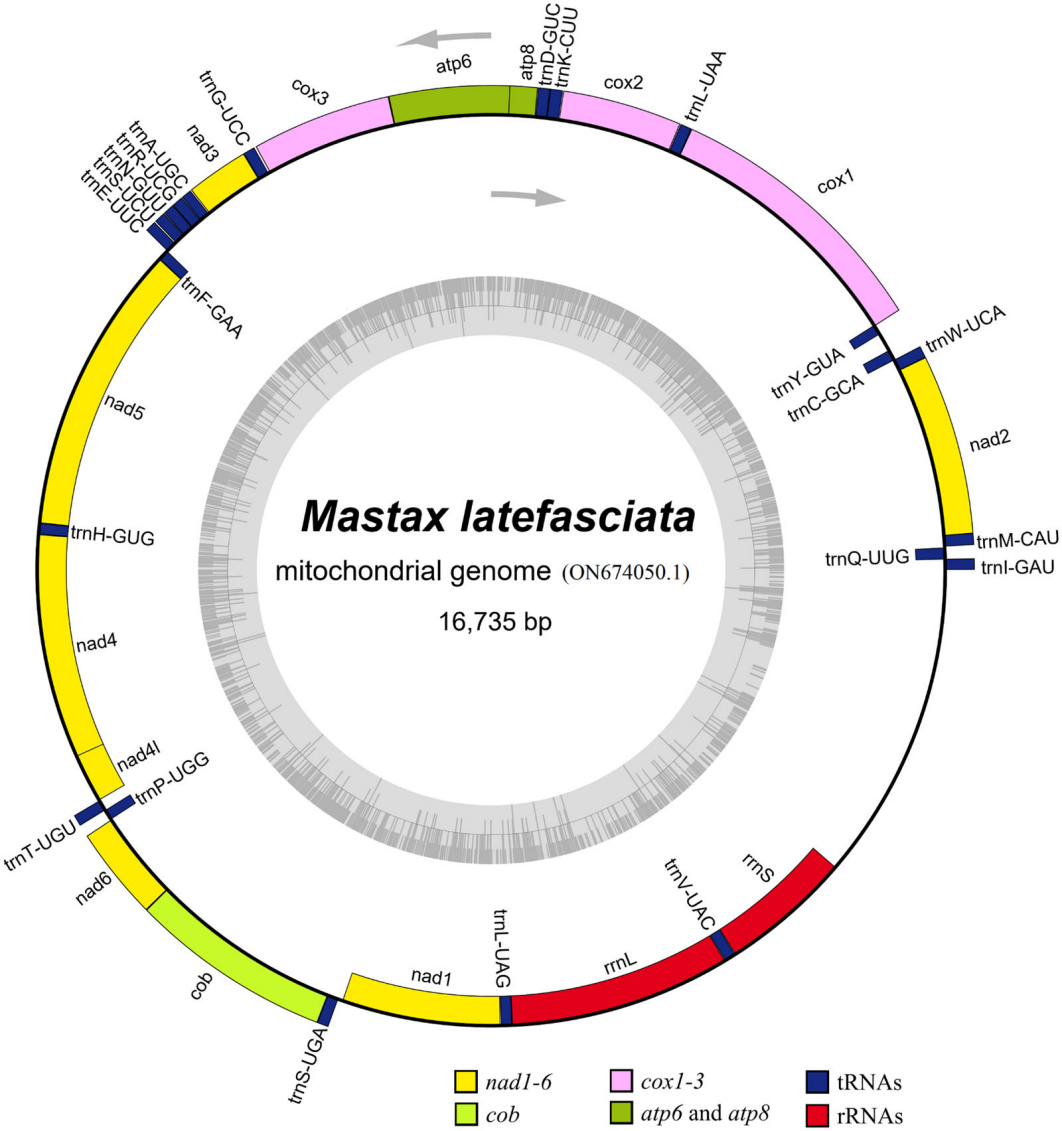
Mitogenome pattern map of *Mastax latefaciata*. Gray arrows indicate 5′→3′; genes inside the circle are transcribed clockwise, genes outside the circle counter clockwise; the grey circle inside was the GC content graph, which marks the 50% threshold.

All 13 PCGs had a typical ATN (Met) start codon, with the exception of *nad1* (a typical TTG start codon): seven PCGs (*nad2*, *cox1*, *atp8*, *nad3*, *nad5*, *nad4l*, and *nad6*) initiated with an ATT start codon; five PCGs (*cox2*, *atp6*, *cox3*, *nad4*, and *cob*) initiated with an ATG start codon. All 13 PCGs contained a typical TAN stop codon: *cob* terminated with a TAG stop codon; seven PCGs (*nad2*, *atp8*, *atp6*, *nad3*, *nad4l*, *nad6*, and *nad1*) ended with a TAA stop codon; five PCGs (*cox1*, *cox2*, *cox3*, *nad5*, and *nad4*) terminated with an incomplete stop codon (T), consisting of a codon that was completed by the addition of A nucleotides at the 3′ end of the encoded mRNA. The 22 tRNA ranged from 61 (trnA-UGC) to 71 bp (trnQ-UUG and trnK-CUU). The rrnL and rrnS were 1319 and 781 bp in length, respectively. The CR, also an AT-rich region, was 1,890 bp in length with an 88.78% AT content and located between the rrnS and trnI-GAU genes.

For phylogenetic analyses, mitogenomes of 16 Carabidae species and two outgroup species [*Morphostenophanes sinicus* (MW853764.1) (Bai et al. 2021) and *Lepisma saccharina* (MT108230.1) (Bai et al. 2020)] were used to evaluate the phylogenetic relationships within the Carabidae using MEGA v11.0.13 (Tamura et al. 2021). The amino acid sequences of 13 PCGs in their mitogenomes were aligned using MEGA (Tamura et al. 2021) with the MUSCLE program (Edgar 2004) using default specifications. The maximum-likelihood (ML) model with the lowest Akaike Information Criterion corrected (AICc) score was considered to be the best. Based on the AICc value (88830.21), general reversible mitochondrial model (mtREV24) with amino acid frequencies (+F), gamma distribution (+ G, parameter = 0.4951, five rate categories) and invariant sites (+I, 22.29% sites) was chosen as the optimal phylogenetic model with 500 bootstrap replications for phylogenetic analysis (Figure 3). The structure of the phylogenetic tree is similar to reported in previous studies (Yu et al. 2019). The phylogenetic position of *Mastax* within the Carabidae was first evaluated using complete mitogenomes, and the results showed that it was close to *Cicindela anchoralis* and *Manticora tibialis*. In this study, the complete mitogenome characteristics of *M. latefasciata* would improve the understanding of the evolution of this species and phylogenetic position of the genus *Mastax* with the related taxa.

**Figure 3.**
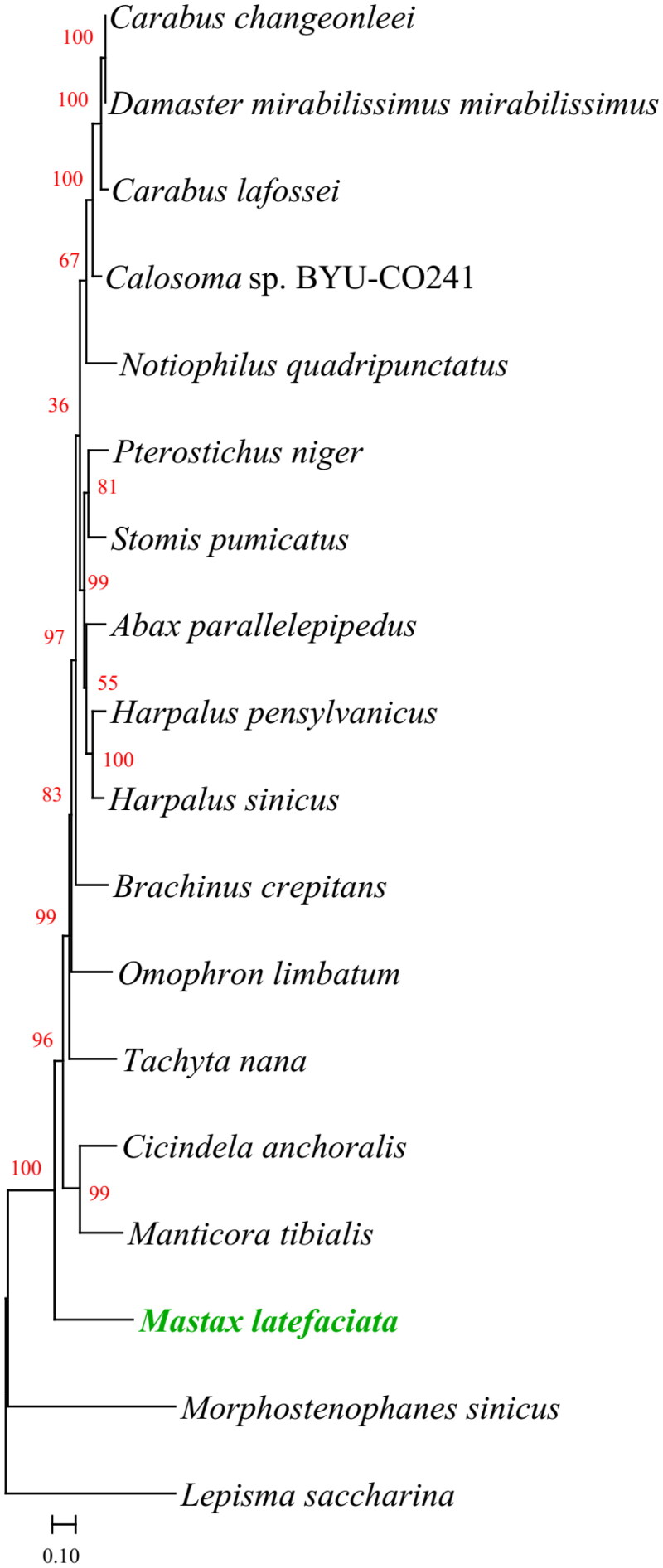
Maximum-Likelihood phylogenetic tree of 18 species based on amino acid sequences of 13 PCGs of their mitogenomes. The highest log-likelihood of the phylogenetic tree was -44748.30. The percentage of trees is shown in red. The complete mitogenome of *M. latefasciata* (ON674050) determined in this study is shown in green. The following sequences were used: *Carabus changeonleei* MG253028 (Wang et al. 2019), *Damaster mirabilissimus mirabilissimus* GQ344500 (Wan et al. 2012), *Carabus lafossei* KY992943 (Liu et al. 2018), *Carabinae* sp. BYU-CO241 GU176340 (Song et al. 2010), *Notiophilus quadripunctatus* MW800883 (Raupach et al. 2022), *Pterostichus niger* KT876909 (Linard et al. 2016), *Stomis pumicatus* KT876914 (Linard et al. 2016), *Abax parallelepipedus* KT876877 (Linard et al. 2016), *Harpalus pensylvanicus* MN245975 (Kieran 2020), *Harpalus sinicus* MN310888 (Yu et al. 2019), *Omophron limbatum* MW800882 (Raupach et al. 2022), *Tachyta nana* KX035142 (Linard et al. 2016), *Cicindela anchoralis* MG253029 (Wang et al. 2018), *Manticora tibialis* MF497821 (López-López and Vogler 2017), *Lepisma saccharina* MT108230 (Bai et al. 2020), and *Morphostenophanes sinicus* (MW853764.1) (Bai et al. 2021).

## Data Availability

The genome sequence data that support the findings of this study are openly available in GenBank of NCBI at https://www.ncbi.nlm.nih.gov under the accession no. ON674050.1. The associated BioProject, Bio-Sample, and SRA numbers are PRJNA857156, SAMN29618062, and SRR20082406 respectively.
